# Supplementary figures and images for: Physiological and Transcriptomic Analyses Reveal Exogenous Trehalose Is Involved in the Responses of Wheat Roots to High Temperature Stress
Source: Plants (Basel). 2021 Dec 1;10(12):2644. doi: 10.3390/plants10122644 (PMC8707964; doi:10.3390/plants10122644)

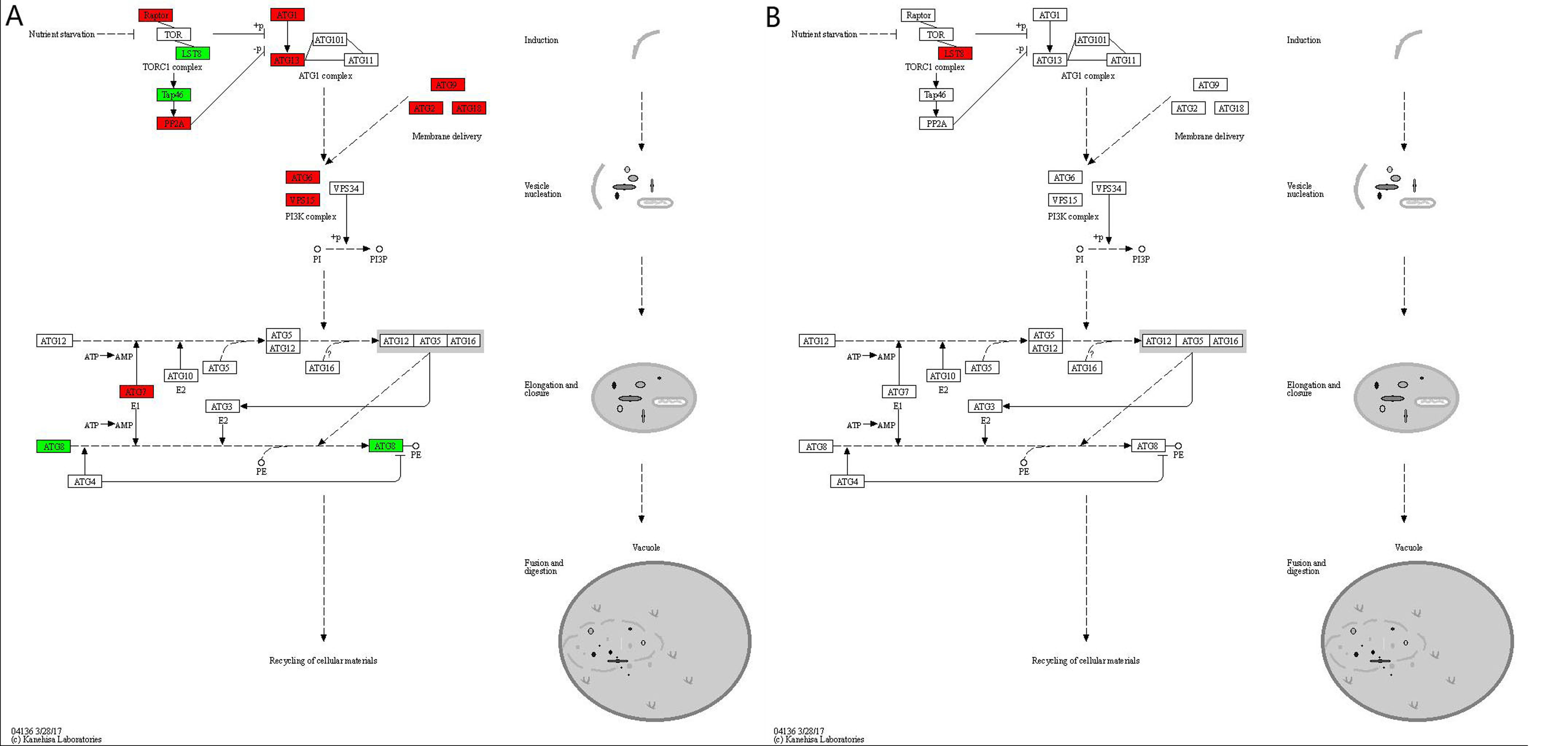

Supplement: Supplementary file 1 [file plants-10-02644-s001.zip › Supplementary materials/Figure S1.jpg]
